# Autoregulation during blood flow restricted exercise offers no additional benefit on thigh muscle hypertrophy and strength adaptations in trained participants: a randomized within-subject 8-week trial

**DOI:** 10.3389/fphys.2026.1772708

**Published:** 2026-02-23

**Authors:** Neslihan Akçay, Nicholas Rolnick, Kadir Keskin, Okan Kamiş, Nevin Köremezli Keskin, Onur Başdemirci, Cem Sofuoğlu, Luke Hughes

**Affiliations:** 1 Faculty of Sport Sciences, Department of Coaching Education, Karabük University, Karabük, Türkiye; 2 The BFR PROS, New York, NY, United States; 3 Faculty of Sports Sciences, Department of Coaching Education, Gazi University, Ankara, Türkiye; 4 Department of Sports and Health, Aksaray University, Aksaray, Türkiye; 5 School of Sport, Exercise and Rehabilitation, Northumbria University, Newcastle upon Tyne, United Kingdom; 6 Faculty of Medicine, Department of Radiology, Karabük University, Karabük, Türkiye; 7 Karabük Training and Research Hospital, Department of Radiology, Karabük, Türkiye

**Keywords:** BFR training, blood flow restriction, hypertrophy, kaatsu, occlusion training, strength, vascular occlusion

## Abstract

**Background:**

With the increasing use of BFR, various cuff design features have emerged, potentially influencing training outcomes. One notable feature is autoregulation, which dynamically modifies tourniquet cuff pressure during muscle contractions to maintain a consistent applied pressure.

**Objective:**

This study investigated the effects of autoregulated versus non-autoregulated blood flow restriction (BFR) training pressures on thigh muscle hypertrophy and strength.

**Methods:**

Using a within-subjects randomized controlled trial design, twenty-one resistance-trained males (≥3 years of experience) completed twice-weekly sessions involving single-leg squats and knee extensions for 8 weeks. One lower limb was trained under autoregulated BFR (AUTO) and the other under non-autoregulated BFR (NONAUTO) conditions using the specific BFR device. Muscle strength (1RM), muscle thickness and cross-sectional area (CSA) of the rectus femoris and vastus lateralis were assessed pre- and post-intervention using ultrasonography. Ratings of perceived exertion (RPE) and discomfort (RPD) were also recorded at the end of weeks 1, 4 and 8.

**Results:**

Both AUTO and NONAUTO conditions led to significant and comparable increases in muscle thickness and CSA across all measured sites of the thigh musculature, as well as improvements in 1RM for both exercises. RPE and RPD scores significantly decreased over time in both conditions, with no between-condition differences. No adverse events occurred in either condition. Autoregulated and non-autoregulated BFR training produced similar muscular hypertrophy and strength adaptations, and perceptual responses.

**Conclusion:**

The current manuscript is the first ever to investigate the chronic effects of BFR training in autoregulated and non-autoregulated applications and is pioneering in this respect. These findings suggest autoregulation does not provide additional physiological benefits under controlled training conditions in the same individual.

## 1 Introduction

Blood flow restriction (BFR) exercise is a technique that involves applying a pneumatic tourniquet cuff to the proximal limb and inflating it to a prescribed percentage of total limb occlusion pressure (LOP) to restrict arterial and venous blood flow (Patterson et al., 2019; Scott et al., 2023). When combined with low-load resistance exercise, BFR enhances muscle hypertrophy and strength gains (Kamiş et al., 2024; Slysz et al., 2016) compared to external-load matched exercise without BFR performed with similar volumes. Some research suggests these benefits may be comparable to those achieved with high-load exercise (de Queiros et al., 2024), making BFR particularly useful for individuals in rehabilitation or those unable to tolerate heavy loads (Hughes et al., 2017).

With the increasing use of BFR during exercise, various cuff design features have emerged, potentially influencing training outcomes (Rolnick, 2024a). One notable feature is autoregulatory pressure adjustment (or described in the BFR training literature as autoregulation), which dynamically modifies tourniquet cuff pressure during muscle contractions to maintain a consistent applied pressure. Accurate autoregulation may facilitate optimization of BFR applications (Hughes et al., 2024) by improving the safety, comfort, and exercise tolerance compared to non-autoregulated cuffs (Jacobs et al., 2023).

However, direct comparisons of autoregulation as an independent variable are limited to three acute studies and a meta-analysis covering 81 studies (Jacobs et al., 2023; Rolnick et al., 2024a; Rolnick et al., 2024b; Clarkson et al., 2024). A recent meta-analysis (Clarkson et al., 2024) found no significant differences in strength, hypertrophy, or functional outcomes between autoregulated and non-autoregulated cuffs applied during exercise. However, these studies did not directly compare the two within the same training program. Therefore, a within-subject study is warranted to quantify the impact of autoregulating applied pressure on relevant training outcomes (e.g., hypertrophy) while minimizing inter-individual variability inherent to between-subject designs. Additionally, it is important to note that the vast majority of the 81 studies included in that meta-analysis did not report essential details regarding BFR apparatus features—such as whether the devices included autoregulation capabilities—or provide any supporting data on pressure accuracy or reliability. This widespread underreporting of critical methodological variables was recently highlighted in a *British Journal of Sports Medicine* editorial, which underscored how such omissions limit reproducibility, safety assessment, and the ability to meaningfully interpret or compare findings across studies (Hughes et al., 2025). In this way, adequate reporting in research studies can further help improve generalizability and reproducibility for practitioners and researchers, especially when BFR cuffs have device features that may impact the magnitude of the BFR stimulus (Rolnick, 2024a; Rolnick et al., 2023; Rolnick, 2024b).

Findings on perceptual responses to BFR exercise remain mixed. While some studies report lower ratings of perceived exertion and discomfort with autoregulated cuffs compared to non-autoregulated devices (Jacobs et al., 2023), others report no meaningful differences (Rolnick et al., 2024a; Rolnick et al., 2024b). These discrepancies may partly reflect differences in device responsiveness (Rolnick, 2024a). For instance, despite both employing autoregulatory technology, the Delfi Personalized Tourniquet System (PTS) and SmartCuffs differ in their pressure modulation algorithms, responsiveness, and mechanical lag. Other BFR systems, such as FitCuffs (FitCuffs BFR Unit, Denmark)—which also feature autoregulatory functionality and have yet to be empirically studied—may offer a more practical and financially accessible solution for many users.

This study aimed to determine whether FitCuffs-enabled autoregulation influences muscular and perceptual responses during an 8-week lower body training program in a within-subject design. A within-subject approach was deliberately selected to isolate the independent effect of pressure regulation while tightly controlling for known sources of inter-individual variability that strongly influence hypertrophy and strength outcomes (e.g., baseline muscle size, fiber-type distribution, vascular characteristics, training history, and perceptual tolerance to BFR). By exposing each participant to both autoregulated and non-autoregulated conditions, this design increases internal validity and statistical sensitivity, allowing smaller between-condition differences to be detected without inflation from between-subject noise. It was hypothesized that the autoregulated condition will produce similar muscle growth in the rectus femoris and vastus lateralis as the non-autoregulated condition, as well as similar muscle strength gains in single-leg squats and knee extensions. Furthermore, it was hypothesized that the autoregulated condition will result in less perceived discomfort and exertion than the fixed-pressure condition at all sampled timepoints.

## 2 Materials and methods

### 2.1 Participants

A medium effect size of f = 0.35 for muscle strength adaptation expected with 8 weeks of BFR training. The primary outcome measure of muscle strength was used to calculate the required sample size *a priori* using G* Power (Version 3.1.9.2). An effect size of f = 0.35 was used for this calculation, based on the expected effect size for improvements in muscle strength with 8 weeks of BFR resistance training. To achieve a power of 80% at an alpha level of 0.05 with a two-way (2 × 2) repeated measures ANOVA, a total of 20 participants were required. To account for potential dropouts, a total of n = 24 participants were recruited. Three participants discontinued due to personal reasons; therefore, a total of 21 male participants completed the study, each with a minimum of 3 years of resistance training experience and a history of engaging in lower-body resistance exercise at least twice per week. Only males were selected to minimize potential sex-based differences in exercise response.

### 2.2 Study design

This study employed a randomized controlled within-subjects design. Participants first completed a survey assessing anthropometrics, current physical activity levels (expressed as hours of participation per week), and inclusion/exclusion criteria to confirm study eligibility. Exercises were performed in the afternoon to minimize the potential influence of circadian rhythms and fatigue on performance (Castelli et al., 2024). Leg dominance was assessed (leg used to kick a ball) and then was randomized to either an autoregulated low-load BFR condition (AUTO) or a fixed pressure, non-autoregulated low-load BFR condition (NONAUTO), utilizing unilateral single-leg squat and knee extension protocols (randomization via http://www.randomizer.org). The other leg received the other condition.

**FIGURE 1:**
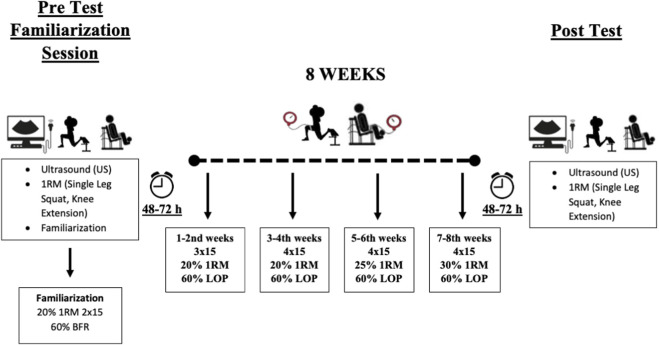
Study design.

All protocols followed a progressive, non-failure resistance training design (1-2nd weeks: 20% 1RM, 60% LOP, 3 × 15 reps; 3-4th weeks: 20% 1RM, 60% LOP, 4 × 15 reps; 5-6th weeks: 25% 1RM, 60% LOP, 4 × 15 reps; 7-8th weeks: 30% 1RM, 60% LOP, 4 × 15 reps) (Figure 1). The ambient temperature was maintained at 21 °C (70°F) for all sessions. Participants were instructed to abstain from caffeine and alcohol for 24 h prior to testing. The study was approved by the university Ethics Committee (approval number: E−77082166–604.01–1095637/2024–1733) and was preregistered on the Open Science Framework (OSF) at https://osf.io/6hj4y/overview?view_only=7baf754c723f43759822a2016700a11b.

Exercises were performed twice weekly over an eight-week period, with 72 h between sessions. A one-minute rest was provided between sets, and a five-minute rest between exercises. During the inter-exercise rest period, the BFR cuffs were deflated. Each session consisted of two exercises—single-leg squats and knee extensions—performed in a consistent sequence across all participants. These exercises targeted the thigh muscles, including the rectus femoris, vastus medialis, and vastus lateralis, and were selected based on their common inclusion in strength and bodybuilding training programs (Haff and Triplett, 2016). The training program followed a progressive overload model to effectively promote gains in strength and muscle hypertrophy (Plotkin et al., 2022). Participants were instructed to refrain from engaging in any additional resistance training during the study period.

### 2.3 Measurements

*Anthropometric Measurements*. The height of each participant was measured with a stadiometer (Seca GmbH & Co. KG, Hamburg, Germany), and body composition analysis was measured by Inbody 270 (Biospace, Seoul, Korea).

*Determination of Limb Occlusion Pressure*. Limb occlusion pressure (LOP) was measured according to manufacturer instructions in the seated position using an automated BFR Unit Device (FitCuffs BFR Unit, Denmark). We choose FitCuffs since it offers practicality and ease of use in training and clinical settings and is marketed as an affordable alternative to more expensive cuff models capable of autoregulation. Participants were comfortably positioned on the leg extension machine, hips positioned at approximately 90˚ flexion, with the cuffs placed off the edge of the seat to prevent artificially elevated LOP readings and increase the specificity of pressure application (Kamis et al., 2024). 60% of LOP was applied consistently during both exercises and exercises were performed for 3 s per repetition. A recent study has provided guidelines for the design and reporting of BFR-related scientific research (Hughes et al., 2025). The relevant cuff characteristics and application parameters used in this study are detailed in Table 1.

**TABLE 1:** Relevant blood flow restriction cuff and protocol characteristics.

Category	
Tourniquet cuff apparatus properties	Reporting element
Manufacturer and model	FitCuffs BFR unit version 4.0
Bladder width	10 cm (4 inches)
Material	Nylon
Type of bladder system	Single-chambered
Shape	Straight
Bladder length	Cuff circumferentially enclosed all participants limbs with bladder applied throughout on the limb
BFR instrument apparatus capabilities	
Manufacturer and model	FitCuffs BFR unit version 4.0
Method of pressure measurement	Automatic via the device
Pressure regulation	Autoregulation and fixed-pressure (depending on randomized leg)
Validity and reliability of limb occlusion pressure measurement	Not reported in the literature
BFR pressure prescription	
Limb occlusion pressure	Refer to Table 2
Posture used for measurement of limb occlusion pressure	Seated
Timings of pressure application	Applied before each exercise including the rest periods and deflated immediately after
Target vs. actual pressure applied	Not reported in the literature

*Muscle Strength*. During the familiarization session, participants underwent a 1RM test for each leg for each of the exercises performed during the trial, beginning with the dominant leg. Participants began testing with a five-minute treadmill warm-up at a self-selected pace. This was followed by a warm-up set of five repetitions at 50% of their self-estimated one-repetition maximum (1RM), then 1–2 additional sets of 2–3 repetitions at approximately 60%–80% of their estimated 1RM. After the warm-up, participants performed single repetitions with progressively increasing loads, with 3- to 5-min rest intervals between attempts. This continued until they were unable to complete a repetition through the full range of motion for both knee extension (90˚ to 0˚ of knee flexion) and single-leg squat (90˚ to 0˚ of knee flexion) exercises. All 1RM tests were performed within five attempts (Haff and Triplett, 2016). Proper technique was closely monitored throughout; any technical deviation rendered the attempt invalid, and the same load had to be successfully lifted before progressing. All trials were supervised by the same qualified strength and conditioning coach to ensure procedural consistency in ensuring proper form throughout the prescribed range of motion.

*Muscle Thickness (Ultrasound) and Muscle Cross-Sectional Area (CSA)*. Sonographic measurements were performed at baseline and following the final training session (week eight), after 48–72 h of rest without physical activity. Musculoskeletal ultrasound assessments were conducted by a physician (COV = 0.2-0.9% across all included measurement sites) blinded to group allocation. Anatomical landmarks, including the greater trochanter and lateral condyles, were used to identify standardized measurement points for muscle thickness and CSA analysis. Proximal-to-distal distances of 30%, 50%, and 70% along the femur were marked for data collection as previously described (Kubo et al., 2011). Measurements were acquired in the axial plane at a 90-degree angle, with the ultrasound probe placed lightly on the skin to avoid compression. At the 30% femoral length, rectus femoris thickness was measured. At both the 50% and 70% levels, the thickness and CSA of the rectus femoris were assessed. The thickness of the vastus lateralis was also evaluated at the 50% and 70% levels. Ultrasound data were collected using a Toshiba Aplio 500 system with a 10 MHz linear probe. Muscle CSA was calculated by automated tracing of the muscle’s inner echoic border, and thickness was defined as the linear distance between the superficial and deep aponeuroses. All measurements were reviewed concurrently by two radiologists, and results were established through mutual consensus.

*Blood Pressure and Heart Rate*. Resting blood pressure and heart rate measurements were performed in the supine position using an automated oscillometric monitor (OMRON M4 Intelli HEM-7155T; Omron Healthcare Co. Ltd., Kyoto, Japan). Following a 5-min rest period at room temperature, two consecutive measurements were taken from the left arm, separated by 1 min. The average of the two values was recorded in mmHg. If the two readings differed by more than 5 mmHg, a third measurement was obtained, and the two closest values were averaged for analysis (Alpert et al., 2023).

*Rating of Perceived Exertion/Discomfort (RPE/RPD)*. The 6–20 Borg scale was used to assess rating of perceived exertion (RPE), while the rating of perceived discomfort (RPD) was evaluated using a scale ranging from 0 (“no discomfort”) to 10 (“maximal discomfort”) (Borg, 1998).

*Autoregulation Perception and Preference*. Participants were asked to identify which leg they believed had undergone autoregulation during the training period. Following the conclusion of the study and disclosure of group assignments, participants were also asked to indicate their preference between the autoregulated and non-autoregulated conditions.

### 2.4 Statistical analysis

All statistical analyses were performed using IBM SPSS Statistics Version 29.0 (IBM Corp., Chicago, IL). Data are presented as mean ± standard deviation, along with 95% confidence intervals. Muscle thickness (mid-belly at 50% and distal belly at 70%) of the vastus lateralis and rectus femoris, as well as one-repetition maximum (1RM) performance in single-leg squat and knee extension, were assessed using a two-way (2 × 2) repeated measures analysis of variance (ANOVA), with factors for condition (autoregulation vs. non-autoregulation) and time (pre-vs. post-intervention). For RPE and RPD outcomes, a two-way repeated measures ANOVA (2 × 3) was conducted to assess differences across sessions 2, 8, and 16 (end of week 1, 4, and 8) between the two conditions. When statistically significant interactions were identified, post-hoc analyses were performed using paired-sample t-tests with Bonferroni correction. Descriptive statistics were also used to report participants’ ability to detect the presence of autoregulation. Statistical significance was set *a priori* at p < 0.05. Effect sizes were calculated using Cohen’s d, defined as the mean difference between two measures divided by the pooled standard deviation. Effect sizes were interpreted as follows: weak (<0.2), weak to moderate (0.2–0.4), moderate (0.4–0.65), moderate to strong (0.65–0.7), and strong (>0.8) (Rubin, 2013).

## 3 Results

All participants completed the exercise sessions, and no adverse events occurred. Participant characteristics are shown in Table 2.

**TABLE 2:** Descriptive characteristics of theparticipants.

Variable	Mean ± SD
Age (year)	20.1 ± 2.1
Height (cm)	179.1 ± 6.9
Weight (kg)	77.6 ± 14.1
BMI (kg/m^2^)	24 ± 3.5
Body fat Mass (kg)	13.2 ± 6.1
Fat free Mass (kg)	64.3 ± 10.4
Skeletal muscle Mass (kg)	36.6 ± 6.3
Percent body fat (%)	16.6 ± 5.6
Resting heart rate (s)	83.71 ± 11.86
Diastolic pressure (mmHg)	70.33 ± 9.21
Systolic pressure (mmHg)	121.1 ± 6.55
LOP-AUTO/LOP-NONAUTO	191.5 ± 14.8/193.5 ± 13.5
60% LOP-AUTO/LOP-NONAUTO	114.9 ± 8.9/115.2 ± 8.8
AUTO right leg (n)	11
AUTO left leg (n)	10

BMI, body mass index; LOP, limb occlusion pressure; AUTO, autoregulation; NONAUTO, non-autoregulation.

### 3.1 Muscle thickness and cross-sectional area

**FIGURE 2:**
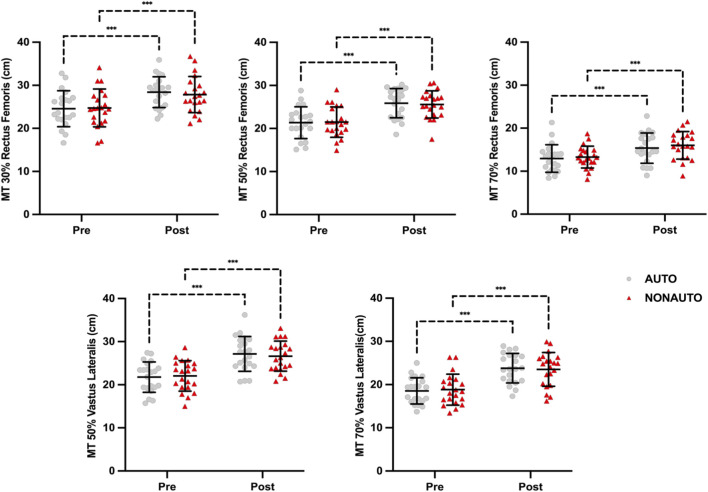
Muscle thickness of the Rectus Femoris and Vastus Lateralis.

*Rectus Femoris 30% MT.* There was a significant main effect of time (*F*_(1,40)_ = 62.26, p < 0.001), indicating an overall increase in muscle thickness from pre-to post-intervention. However, the main effect of condition (AUTO vs. NONAUTO) was not significant (*F*_(1,40)_ = 0.025, p = 0.875), nor was the interaction effect between time and condition (*F*_(1,40)_ = 0.704, p = 0.406). The AUTO condition demonstrated a 14.6% increase in RF muscle thickness compared to a 14.1% increase in the NONAUTO condition (Table 3; Figure 2).

**TABLE 3:** Muscle thickness and CSA of the RF and VL muscles.

	Pre	Post	Mean difference	Cohen’s d	ANOVA
Muscle thickness (mm)					
30% rectus femoris AUTO NONAUTO	24.80 ± 4.49 24.41 ± 4.90	28.41 ± 3.57 27.85 ± 4.21	3.84 ± 2.91 (95% CI: 2.59–5.08) 3.10 ± 2.78 (95% CI: 1.91–4.29)	0.95 0.68	Group x time: *F*_(1,40)_ = 0.704, p = 0.406 Group: *F*_(1,40)_ = 0.025, p = 0.875 Time: *F*_(1,40)_ = 62.26, p < 0.001
50% rectus femoris AUTO NONAUTO	20.70 ± 2.51 20.56 ± 2.51	25.89 ± 3.40 25.60 ± 3.17	4.55 ± 3.00 (95% CI: 3.27–5.83) 4.10 ± 2.36 (95% CI: 3.09–5.12)	1.52 1.44	Group x time: *F*_(1,40)_ = 0.282, p = 0.598 Group: *F*_(1,40)_ = 0.005, p = 0.940 Time: *F*_(1,40)_ = 107.9, p < 0.001
70% rectus femoris AUTO NONAUTO	12.56 ± 2.26 13.71 ± 2.35	15.38 ± 3.52 16.01 ± 3.20	2.42 ± 2.20 (95% CI: 1.48–3.36) 2.73 ± 2.19 (95% CI: 1.79–3.67)	0.82 0.97	Group x time: *F*_(1,40)_ = 0.203, p = 0.655 Group: *F*_(1,40)_ = 0.281, p = 0.599 Time: *F*_(1,40)_ = 57.89, p < 0.001
50% vastus lateralis AUTO NONAUTO	20.70 ± 2.90 21.05 ± 4.06	27.16 ± 4.04 26.62 ± 3.49	5.38 ± 2.64 (95% CI: 4.25–6.51) 4.56 ± 3.11 (95% CI: 3.23–5.89)	1.53 1.21	Group x time: *F*_(1,40)_ = 0.836, p = 0.366 Group: *F*_(1,40)_ = 0.016, p = 0.900 Time: *F*_(1,40)_ = 124.5, p < 0.001
70% vastus lateralis AUTO NONAUTO	19.41 ± 2.96 18.33 ± 2.11	23.80 ± 3.42 23.52 ± 3.89	5.24 ± 3.50 (95% CI: 3.74–6.73) 4.67 ± 3.03 (95% CI: 3.38–5.97)	1.64 1.49	Group x time: *F*_(1,40)_ = 0.314, p = 0.578 Group: *F*_(1,40)_ = 0.00005, p = 0.994 Time: *F*_(1,40)_ = 96.38, p < 0.001
Muscle CSA (cm^2^)					
50% rectus femoris AUTO NONAUTO	10.16 ± 1.95 9.70 ± 1.69	12.45 ± 2.48 12.20 ± 2.52	2.25 ± 1.67 (95% CI: 1.54–2.97) 1.88 ± 1.16 (95% CI: 1.39–2.38)	1.01 0.88	Group x time: *F*_(1,40)_ = 0.691, p = 0.411 Group: *F*_(1,40)_ = 0.008, p = 0.928 Time: *F*_(1,40)_ = 86.59, p < 0.001
70% rectus femoris AUTO NONAUTO	3.59 ± 1.08 3.81 ± 0.77	4.28 ± 1.32 4.23 ± 1.00	0.73 ± 0.69 (95% CI: 0.44–1.03) 0.71 ± 0.66 (95% CI: 0.43–0.99)	0.61 0.79	Group x time: *F*_(1,40)_ = 0.016, p = 0.899 Group: *F*_(1,40)_ = 0.012, p = 0.910 Time: *F*_(1,40)_ = 48.10, p < 0.001

*Rectus Femoris 50% MT.* There was a significant main effect of time (*F*_(1,40)_ = 107.9, p < 0.001), indicating a substantial increase in muscle thickness from pre-to post-intervention. However, the main effect of condition was not significant (*F*_(1,40)_ = 0.0056, p = 0.940), nor was the interaction effect between time and condition (*F*_(1, 40)_ = 0.282, p = 0.598). The AUTO condition demonstrated a 25.1% increase in RF 50% muscle thickness compared to a 24.5% increase in the NONAUTO condition (Table 3; Figure 2).

*Rectus Femoris 70% MT.* There was a significant main effect of time (*F*_(1,40)_ = 57.89, p < 0.001), indicating a meaningful increase in muscle thickness from pre-to post-intervention. However, the main effect of condition was not significant (*F*_(1,40)_ = 0.281, p = 0.599), nor was the interaction between time and condition (*F*_(1,40)_ = 0.203, p = 0.655). The AUTO condition demonstrated an 22.5% increase in RF 70% muscle thickness compared to a 16.8% increase in the NONAUTO condition (Table 3; Figure 2).

**FIGURE 3:**
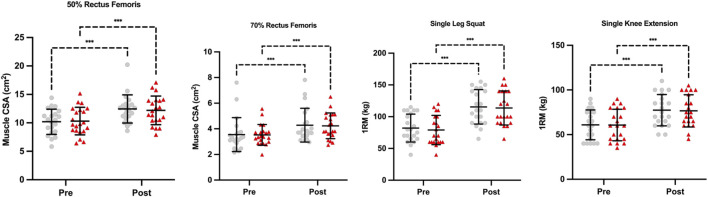
Muscle cross-sectional area and muscle strength performance.

*Rectus Femoris 50% CSA.* There was a significant main effect of time (*F*_(1,40)_ = 86.59, p < 0.001), indicating a substantial increase in CSA from pre-to post-intervention. However, neither the main effect of condition (*F*_(1,40)_ = 0.008, p = 0.928) nor the interaction effect between time and condition (*F*_(1,40)_ = 0.69, p = 0.411) was statistically significant. The AUTO condition demonstrated a 22.5% increase in RF CSA compared to an 25.8% increase in the NONAUTO condition (Table 3; Figure 3).

*Rectus Femoris 70% CSA.* There was a significant main effect of time (*F*_(1,40)_ = 48.1, p < 0.001), indicating a meaningful increase in CSA from pre-to post-intervention. However, the main effect of condition (*F*_(1,40)_ = 0.012, p = 0.910) and the interaction effect between time and condition (*F*_(1,40)_ = 0.016, p = 0.899) were not statistically significant. The AUTO condition demonstrated a 19.2% increase in RF CSA compared to a 11.0% increase in the NONAUTO condition (Table 3; Figure 3).

*Vastus Lateralis 50% MT.* There was a significant main effect of time (*F*_(1,40)_ = 124.5, p < 0.001). However, the main effect of condition (*F*_(1,40)_ = 0.016, p = 0.900) and the interaction effect were not significant (*F*_(1,40)_ = 0.836, p = 0.366). The AUTO condition demonstrated a 31.2% increase in RF CSA compared to a 26.5% increase in the NONAUTO condition (Table 3; Figure 2).

*Vastus Lateralis 70% MT.* There was a significant main effect of time (*F*_(1,40)_ = 96.38, p < 0.001). However, the main effect of condition (*F*_(1,40)_ = 0.00006, p = 0.994) and the interaction effect were not significant (*F*_(1,40)_ = 0.314, p = 0.578). The AUTO condition demonstrated a 22.6% increase in RF CSA compared to a 28.3% increase in the NONAUTO condition (Table 3; Figure 2).

### 3.2 Muscle strength

*1RM Single Leg Squat.* There was a significant main effect of time (*F*_(1,40)_ = 507.1, p < 0.001). However, the main effect of condition (*F*_(1,40)_ = 0.1003, p = 0.753) and the interaction effect (time X condition) (*F*_(1,40)_ = 0.2232, p = 0.639) were not significant (Table 4; Figure 3). The AUTO condition demonstrated a 40.6% increase compared to a 43.9% increase in the NONAUTO condition.

**TABLE 4:** Muscle strength performance.

	Pre	Post	Mean difference	Cohen’s d	ANOVA
1RM (kg)					
Single leg squat AUTO NONAUTO	82.14 ± 22.00 79.05 ± 23.11	115.48 ± 27.06 113.81 ± 26.83	33.33 ± 9.79 (95% CI: 29.15–37.52) 34.76 ± 9.81 (95% CI: 30.57–38.96)	1.35 1.39	Group x time: *F*_(1,40)_ = 0.223, p = 0.639 Group: *F*_(1,40)_ = 0.10, p = 0.753 Time: *F*_(1,40)_ = 507.1, p < 0.001
Single knee extension AUTO NONAUTO	60.95 ± 16.70 60.71 ± 17.70	77.38 ± 17.51 76.67 ± 18.05	16.43 ± 5.28 (95% CI: 14.17–18.69) 15.95 ± 4.90 (95% CI: 13.86–18.05)	0.96 0.89	Group x time: *F*_(1,40)_ = 0.091, p = 0.764 Group: *F*_(1,40)_ = 0.007, p = 0.929 Time: *F*_(1,40)_ = 424.2, p < 0.001

*1RM Single Knee Extension.* There was a significant main effect of time (*F*_(1,40)_ = 424.2, p < 0.001). However, the main effect of condition (*F*_(1,40)_ = 0.008, p = 0.929) and the interaction effect (time X condition) (*F*_(1,40)_ = 0.09, p = 0.764) were not significant (Table 4; Figure 3). The AUTO condition demonstrated a 26.9% increase compared to a 26.3% increase in the NONAUTO condition.

### 3.3 Perceptual responses and detection of autoregulation and preference

**FIGURE 4:**
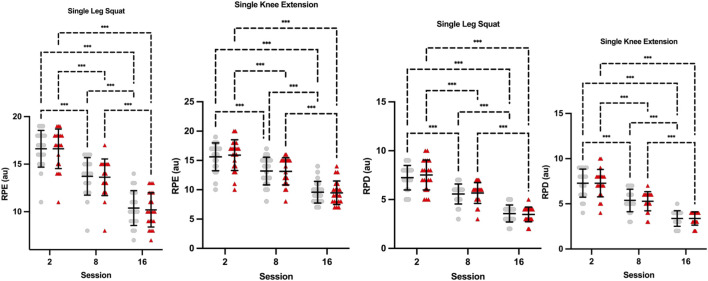
Perceptual responses to single leg squat and single knee extension across sessions *Knee Extension RPE.* There was a significant main effect of time (*F*_(2,80)_ = 156.0, p < 0.001). However, the main effect of condition (*F*_(1,40)_ = 0.007, p = 0.934) and the interaction effect (time X condition) (*F*_(2,80)_ = 0.171, p = 0.843) were not significant (Figure 4).

*Single-leg squat RPD.* There was a significant main effect of time (*F*_(2,80)_ = 302.9, p < 0.001). However, the main effect of condition (*F*_(1,40)_ = 0.106, p = 0.747) and the interaction effect (time X condition) (*F*_(2,80)_ = 0.7368, p = 0.482) were not significant (Figure 4).

*Single-leg squat RPE.* There was a significant main effect of time (*F*_(2,80)_ = 228.7, p < 0.001). However, the main effect of condition (*F*_(1,40)_ = 0.384, p = 0.846) and the interaction effect (time X condition) (*F*_(2,80)_ = 0.052, p = 0.950) were not significant (Figure 4).

*Knee Extension RPD.* There was a significant main effect of time (*F*_(2,80)_ = 268.2, p < 0.001). However, the main effect of condition (*F*_(1,40)_ = 0.01008, p = 0.921) and the interaction effect (time X condition) (*F*_(2,80)_ = 0.05, p = 0.948) were not significant (Figure 4).

*Detection of Autoregulation and Preference.* Participants’ preference for the NONAUTO regulation mode increased over time, whereas preference for the AUTO regulation mode decreased. As the trial progressed, participants were able to correctly identify the regulation mode.

## 4 Discussion

This study is the first to compare muscular adaptations, strength performance and perceptual responses between an autoregulated (AUTO) and a fixed-pressure, non-autoregulated (NONAUTO) lower-body training program. Our findings indicate that both conditions led to significant increases in muscle thickness (as assessed by ultrasonography) and thigh CSA, with no significant differences between groups. Strength gains in both single-leg squats and knee extensions significantly increased pre-to-post intervention, again with no differences between AUTO and NONAUTO. Perceptual responses, including ratings of perceived discomfort (RPD) and exertion (RPE), significantly decreased over time in both conditions, with no between-group differences. These findings suggest that autoregulation does not notably influence muscular growth, strength adaptations, or perceptual responses compared to a structured, non-autoregulated approach, which is in alignment with a recent systematic review and meta-analysis (Clarkson et al., 2024).

Importantly, the AUTO and NONAUTO conditions differed only in pressure regulation mode; all other key training determinants were standardized (relative pressure prescription, exercise selection, volume progression, repetition cadence, and rest periods). Under these controlled conditions, the absence of between-condition differences in our cohort suggests that when BFR training pressure is prescribed relative to limb occlusion pressure and training variables are tightly matched, dynamic pressure modulation does not appear to meaningfully augment the stimulus for hypertrophy, strength, or perceptual experiences beyond a fixed-pressure application. Practically, this indicates that the primary drivers of adaptation in this context were likely the progressive, periodized training program and BFR exposure *per se*, rather than the pressure-regulation strategy.

### 4.1 Muscular outcomes

The current study found no significant differences in muscle hypertrophy outcomes over 8 weeks between the AUTO and NONAUTO conditions. These findings align with the meta-analytic results of Clarkson et al. (2024), who also reported comparable hypertrophy outcomes between autoregulated and unregulated BFR cuff systems (Clarkson et al., 2024). Notably, our study extends these findings by directly comparing AUTO and NONAUTO conditions within the same participants—an approach not represented in the studies included in Clarkson et al.’s meta-analysis. Clarkson et al. (2024) reported muscle thickness increases ranging from 2% to 18% with autoregulated cuffs and 1%–13% with unregulated cuffs (n = 23), which largely matches the ranges observed in our study in the rectus femoris MT (14.1%–25.1%). and vastus lateralis MT (22.6%–31.2%) (Clarkson et al., 2024). Similarly, they found muscle cross-sectional area (CSA) increased by 2%–24% in the autoregulated condition and 2%–23% in the unregulated condition (n = 29), which is also consistent with our findings in the rectus femoris (11.0%–25.8%). These parallel results further support the conclusion that autoregulation may not confer additional benefits for muscular adaptations under well-controlled training conditions.

The findings on muscle strength also align with those of Clarkson et al. (2024), while expanding upon them through a within-subject design. Clarkson et al. reported that in studies assessing 1RM strength (n = 39), autoregulated BFR systems led to strength gains ranging from 3% to 32%, while unregulated systems showed gains between 6% and 55%, with no significant differences between groups (Clarkson et al., 2024). Our study demonstrated comparable improvements, with knee extension 1RM increasing by 26.3% (NONAUTO) and 26.9% (AUTO). Similarly, single-leg squat 1RM increased by 40.59% (AUTO) – 43.95% (NONAUTO), which falls largely within the ranges reported by Clarkson et al. (2024), further supporting the conclusion that both BFR pressure applications produce robust strength gains.

Autoregulation is theorized to influence muscular adaptations such as hypertrophy and strength by reducing contraction-induced pressure drift and thereby maintaining a more consistent restriction stimulus across repetitions (Rolnick, 2024a; Jacobs et al., 2023). However, several factors may have minimized the opportunity for autoregulation to confer an advantage in the present study. First, pressure was prescribed relative to individual LOP (60% LOP) in both conditions, which already individualizes the stimulus and may reduce variability that autoregulation is intended to address. Second, the protocol used a controlled repetition cadence (3s/contraction) and a progressive, non-failure model, which may limit large intra-set hemodynamic swings and reduce the practical impact of dynamic pressure regulation. Third, participants were resistance-trained, potentially lowering inter-individual variability in training responsiveness compared with untrained cohorts. Collectively, these factors may explain why comparable hypertrophy and strength adaptations were observed despite differences in pressure regulation mode.

### 4.2 Perceptual outcomes and detection of autoregulation and preference

Hughes et al. (2018) investigated the acute perceptual responses to three different BFR devices and concluded that the autoregulated personalized tourniquet device better managed perceptual responses compared to an unregulated cuff inflated to the same percentage of occlusion pressure (Hughes et al., 2018). Our findings contrast, showing no differences in ratings of perceived exertion or discomfort between autoregulated and unregulated conditions during either exercise or at any time point measured, although we did see a reduction in perceptual demands over time from high RPE/RPD to low RPE/RPD (Figure 4). As this is the first longitudinal investigation into the impact of autoregulation, we can only compare with existing research on BFR exercise. In agreement with prior research (Martín-Hernández et al., 2017; Mattocks et al., 2019), perceptual demands appear to diminish over time, even with adjustments of training loads, possibly due to familiarity with the BFR stimulus. However, our research adds to this observation that the presence of autoregulation does not diminish these perceptual demands further. As for the detection of autoregulation and preferences, participants’ preference for the NONAUTO regulation mode increased over time (from 52% at week 1%–81% at week 8), whereas preference for the AUTO regulation mode decreased (from 48% to 19%). We speculate that this shift may be attributable to differences in the perceived consistency of applied pressure. Fixed-pressure BFR may have been experienced as more stable and predictable across repetitions, whereas the dynamic pressure adjustments inherent to autoregulation could have been perceived as less consistent or more distracting. Such fluctuations may have been interpreted by participants as a weaker or less effective stimulus, despite no objective differences in training outcomes. Future studies should directly quantify the magnitude and temporal characteristics of interface pressure fluctuations in FitCuffs during exercise, as commercially available BFR systems have been shown to differ substantially in their ability to maintain consistent pressure throughout a training bout (Hughes et al., 2024; Rolnick et al., 2025a). In week 1, 71% of participants correctly identified the regulation mode, while 29% guessed incorrectly. By week 4, all participants correctly identified the regulation mode, and by week 8, 95% identified it correctly, while 5% guessed incorrectly.

Acute studies have reported mixed perceptual findings across autoregulated systems, suggesting that ‘autoregulation’ is not a uniform exposure and may depend on device responsiveness, control algorithms, and the magnitude/timing of pressure adjustments during contractions (Rolnick, 2024a; Jacobs et al., 2023; Rolnick et al., 2024a; Rolnick et al., 2024b). In the current study, perceptual demands decreased over time in both conditions, consistent with habituation to the BFR stimulus (Mattocks et al., 2019). This habituation may have attenuated any early-session perceptual distinctions between conditions, particularly because RPE/RPD were sampled at discrete time points rather than continuously within sets where pressure adjustments may be most noticeable. Therefore, the lack of perceptual differences should be interpreted as evidence that autoregulation did not provide an additional comfort advantage in this training context, not that perceptual differences are impossible across all devices or protocols, particularly as comfort levels appear to differ depending on cuff type and/or presence of autoregulation (Rolnick, 2024a; Jacobs et al., 2023; Rolnick et al., 2024c; Rolnick et al., 2025b).

### 4.3 Limitations

This study is the first to examine chronic training outcomes using autoregulation in BFR, though several limitations must be acknowledged. Our primary limitation is that the FitCuffs device was used to deliver the autoregulatory stimulus; however, unlike the empirically validated Delfi Personalized Tourniquet System, FitCuffs lacks evidence for its responsiveness and stimulus (Hughes et al., 2018). It remains unclear whether device responsiveness affects the magnitude of BFR stimulus and resulting adaptations or if the adaptations would differ with slower or faster repetition cadences over a training period (Rolnick, 2024a). Future research should consider validated devices like Delfi Personalized Tourniquet System, which has shown superior pressure maintenance to other commercially available BFR devices (Hughes et al., 2024). Prior findings suggest autoregulation may reduce post-exercise arterial stiffness (Rolnick et al., 2024b; Rolnick et al., 2024c), a possible safety benefit warranting further investigation. Though no adverse events were observed, underlying arterial differences between autoregulated and non-autoregulated conditions remain unknown. Second, the use of a non-failure, fixed-rep scheme may have also limited autoregulation’s impact, though previous research shows similar outcomes between failure and non-failure training (Sieljacks et al., 2019). Third, assigning autoregulation based on leg dominance may have introduced bias due to potential baseline differences in neural drive, strength, or motor control between dominant and non-dominant limbs. Although this design increases internal validity by controlling for inter-individual variability, leg dominance could systematically influence training adaptations, perceptual responses, or performance outcomes, independent of the pressure regulation mode. Future studies may consider counterbalancing autoregulation across leg dominance or matching limbs based on baseline strength or hypertrophy to better isolate the effects of autoregulation. Moreover, due to limitations in the imaging field, CSA measurements for the vastus lateralis were not feasible and echo intensity analyses could not be performed, as only representative ultrasound images were recorded on the computer. Future studies could include echo intensity measurements. Thus, future studies should incorporate vastus lateralis CSA to elucidate the possibility of muscle-specific responses to AUTO. We acknowledge that our muscular responses are on the higher end of the reported BFR literature (Clarkson et al., 2024) and that we cannot conclusively rule out that we captured some muscle edema as part of our assessments. However, we attempted to control for oedema by delaying imaging assessments 48–72 h as well as using a blinded examiner with excellent COV. Finally, findings should not be generalized beyond healthy, resistance-trained individuals.

### 4.4 Practical takeaways

Autoregulated BFR cuffs are often marketed as enhancing the safety and effectiveness of BFR training by dynamically adjusting pressure during exercise. However, results from this 8-week within-subject trial do not support these claims in healthy, resistance-trained males. Both the AUTO and NONAUTO conditions produced equivalent improvements in muscle hypertrophy, strength, and perceptual responses, with no adverse events in either group. These findings suggest that clinicians and practitioners do not require cuffs with autoregulatory features to achieve optimal BFR outcomes, provided that training protocols adhere to established safety and loading guidelines. For most healthy, trained individuals, device simplicity, accessibility, and ease of use may be prioritized over autoregulation without compromising effectiveness.

## 5 Conclusion

Previous studies have reported mixed findings on whether autoregulated BFR pressure provides meaningful efficacy or safety benefits compared with fixed-pressure (non-autoregulated) BFR, and all prior research has focused only on acute responses. In this study, muscle strength and hypertrophy adaptations from single-leg squats and knee extensions significantly improved from pre-to post-intervention, with no differences between autoregulated and non-autoregulated conditions using the FitCuffs BFR cuff. Perceptual responses also improved similarly across both conditions, with no adverse events reported. This is the first study to compare FitCuffs-enabled autoregulation with a fixed-pressure application within subjects over a multi-week resistance training protocol, ensuring high internal validity. Together, these findings suggest that when BFR pressure is prescribed relative to LOP and training variables are standardized, pressure regulation strategy alone is unlikely to be a primary determinant of chronic adaptation in trained individuals. However, evidence on the accuracy and responsiveness of the autoregulatory function is lacking and future research is needed to determine its intrinsic capacity to maintain a consistent set/interface pressure. For coaches, clinicians, and rehabilitation professionals, ease of use and logistical considerations may be more important than autoregulatory features when selecting BFR equipment. Further research should explore the role of autoregulation in other BFR devices and in populations with lower training tolerance or clinical conditions.

## Data Availability

The raw data supporting the conclusions of this article will be made available by the authors, without undue reservation upon reasonable request.
